# Clinical, imaging, and neuropathological characterization of multiple system degeneration associated with a novel *SERAC1* variant in a mixed-breed dog

**DOI:** 10.1093/jvimsj/aalag141

**Published:** 2026-07-13

**Authors:** Tania Al Kafaji, Çağla Aytaş, Assami-Carina Perret, Vidhya Jagannathan, Tosso Leeb, Carlo Cantile, Antonella Gallucci

**Affiliations:** Veterinary Neurologic Center “La Fenice”, 09047 Selargius, Italy; Department of Veterinary Sciences, University of Pisa, 56124 Pisa, Italy; Institute of Genetics, Vetsuisse Faculty, University of Bern, 3001 Bern, Switzerland; Graduate School for Cellular and Biomedical Sciences, University of Bern, 3012 Bern, Switzerland; Institute of Genetics, Vetsuisse Faculty, University of Bern, 3001 Bern, Switzerland; Institute of Genetics, Vetsuisse Faculty, University of Bern, 3001 Bern, Switzerland; Department of Veterinary Sciences, University of Pisa, 56124 Pisa, Italy; Veterinary Neurologic Center “La Fenice”, 09047 Selargius, Italy

**Keywords:** brain disease, genetic disease, neurodegenerative disease, neuropathology

## Abstract

A 7-month-old spayed female mixed-breed dog was evaluated for a subacute, progressive, cerebellar syndrome characterized by ataxia and intention tremors. Brain magnetic resonance imaging (MRI) revealed moderate cerebellar atrophy and mild bilateral symmetrical intra-axial lesions at the level of the caudate nuclei. A neurodegenerative disorder was suspected. Over a 2-year period, signs of neurologic disease worsened with suspected myoclonic epileptic seizures and severe cerebellar ataxia. Follow-up MRI showed progressive cerebellar and cerebral atrophy, as well as well-defined, bilateral, and symmetrical lesions affecting the caudate nuclei. Histopathology revealed severe cerebellar degeneration with a loss of Purkinje cells and depletion of the granular and molecular layers. Malacic areas at the level of the caudate nuclei characterized by extensive necrosis were observed. Genetic testing identified a clear top candidate variant in the *SERAC1* gene on chromosome 1. These findings are consistent with multiple system degeneration, a rare inherited neurodegenerative disorder resembling MEGD(H)EL syndrome (3-methylglutaconic aciduria with deafness-dystonia, [hepatopathy], encephalopathy, and Leigh-like syndrome) in humans.

A 7-month-old, spayed, female, mixed-breed dog was examined because of a 1-month history of subacute and progressive onset of 4-limb incoordination and head tremors. The physical examination did not detect any abnormalities. Neurological examination revealed a normal mental status and behavior, mild intention head tremors, cerebellar ataxia characterized by a hypermetric gait in all limbs, and a decreased bilateral menace response (Supplementary material, Video S1). Hypermetric hopping responses were also observed. The remainder of the neurological examination was normal. These findings were consistent with a cerebellar disorder.

The diagnostic workup included a biochemistry profile, CBC, blood ammonia measurement, brain magnetic resonance imaging (MRI), and cerebrospinal fluid (CSF) analysis. Biochemistry, CBC, and blood ammonia concentrations were within reference ranges. Brain MRI was performed using an open magnet Airis II 0.3 T with fast spin-echo (FSE) T2-weighted (T2W) sagittal and transverse sequences, dorsal T2W fluid-attenuated inversion recovery (FLAIR), and T1-weighted (T1W) sequences in all 3 planes before and after intravenous gadolinium administration. Magnetic resonance imaging revealed reduced cerebellar volume with deepening of the cerebellar sulci and CSF accumulation between the cerebellar folia, which was more evident in the dorsorostral aspect of the cerebellum. Mild and poorly visible bilateral and symmetric intra-axial lesions at the level of the caudate nuclei were observed. They were characterized by smooth margins and an irregular shape, with mild T2W and FLAIR hyperintensity and T1W isointensity without contrast enhancement. Cerebrospinal fluid collected from the cisterna magna had a total nucleated cell count and protein concentrations within normal limits. A neurodegenerative disorder (including neurometabolic disease) mainly involving the cerebellum and caudate nuclei was primarily suspected.

After 2 years, the dog returned with acute episodes characterized by disorientation, hypersalivation, myoclonic movements of the head and trunk, and urinary incontinence. These episodes lasted 1-2 min followed by spontaneous recovery. About 15 episodes were reported during the day each separated by normal interictal periods. Clinical signs were compatible with focal epileptic seizures. Movement disorders were considered less likely due to the presence of autonomic signs and behavioral changes. In addition to the previously described signs of cerebellar dysfunction, the dog exhibited disorientation, an absence of the bilateral menace response, and decreased postural reactions on paw replacement tests in all 4 limbs. Neurological findings were consistent with an intracranial multifocal neurolocalization involving both the forebrain and cerebellum (Video S2). A progressive degenerative disease or an infectious/inflammatory process superimposed on a preexisting condition was suspected.

Follow-up brain MRI was performed using the open magnet Airis II with FSE T2W sagittal and transverse sequences, dorsal FLAIR, and T1W sequences in all 3 planes before and after administration of intravenous contrast. The study revealed mild widening of the cerebral sulci outlining the gyri, with secondary CSF accumulation, interthalamic adhesion size reduction, and enlargement of the fourth ventricle. Compared to the previous MRI, there was progressive cerebellar volume loss with further deepening of the cerebellar sulci (Figure 1). The previously described lesions at the level of the caudate nuclei were markedly increased in size and appeared oval-shaped with well-defined margins, showing T2W and FLAIR hyperintensity and T1W hypointensity without contrast enhancement (Figure 2). Cerebrospinal fluid collected from the cisterna magna was normal. Magnetic resonance imaging findings were consistent with an encephalopathy involving both caudate nuclei, with severe cerebellar and mild cerebral atrophy, suggesting a neurodegenerative disorder as the primary differential diagnosis.

**Figure 1:**
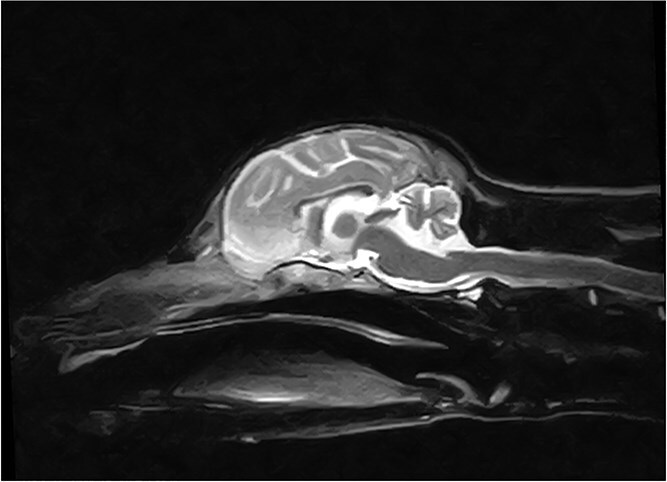
Sagittal T2-weighted image sequence. Decreased cerebellar size with deepening of the cerebellar sulci. Widening of cerebral sulci outlining the gyri, with secondary CSF accumulation, interthalamic adhesion size reduction, and enlargement of the fourth ventricle. Abbreviation: CSF = cerebrospinal fluid.

**Figure 2:**
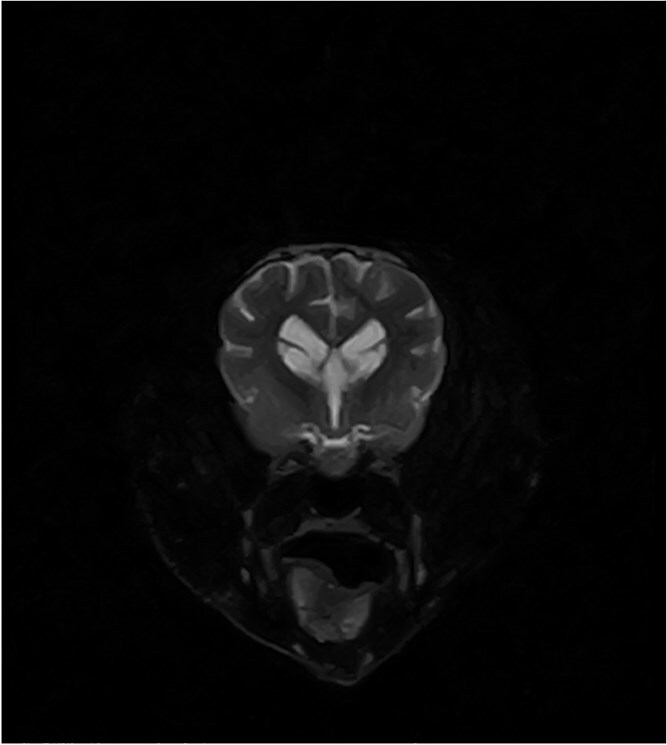
Transverse T2W image at the level of the caudate nuclei. There are bilateral and symmetrical, intra-axial T2W hyperintense lesions at the level of the caudate nuclei, characterized by oval shape and well-defined margins. Abbreviation: T2W = T2-weighted.

Medical treatment was initiated with phenobarbital (2.5 mg/kg PO q12h), and acetylcarnitine (50 mg/kg PO q24h). No further myoclonic episodes were reported, although neurological signs remained stable. After 7 months, the clinical signs worsened and the dog developed a loss of balance and frequent falling to either side. Bilateral vision loss was also noted, and intention tremors increased. Hypertonia and spasticity were present in all 4 limbs, predominantly in the thoracic limbs (Supplementary material, Video S2). Since a mitochondrial disorder was the most likely differential, thiamine, coenzyme Q, and a supplement containing L-lysine, taurine, L-arginine, methionine, B-complex vitamins, vitamin E, zinc, and selenium were added to the therapy. A ketogenic diet was also introduced.

Despite treatment, clinical signs progressively worsened, resulting in the inability to maintain a standing position and generalized limb hypertonia. Due to a poor quality of life, euthanasia was elected at about 3 years of age. The brain was removed, fixed in 10% neutral buffered formalin, and submitted to the Neuropathology Section of the Department of Veterinary Science of the University of Pisa. Transverse sections of the fixed brain showed bilateral malacic areas at the level of the caudate nuclei and, multifocally, at the boundary between the cortical gray matter and subcortical white matter (Figure 3) in the frontal, parietal, and piriform lobes. The cerebellum appeared uniformly reduced in volume with atrophy of the folia (Figure 4). The tissue was routinely processed, embedded in paraffin, and 4-μm-thick histological sections were stained with hematoxylin and eosin and Luxol fast blue. Immunohistochemistry was performed on selected sections using the avidin–biotin–peroxidase complex method. Antigen retrieval was carried out using steam heat. Primary antibodies against the following antigens were used: glial fibrillary acidic protein (1:250, rabbit polyclonal, Dako, Carpinteria, CA, USA), Iba1 (1:1000, rabbit polyclonal, Fujifilm Wako Pure Chemical Corporation, Osaka, Japan), and parvalbumin (1:1000, mouse monoclonal, clone PARV-19, Sigma-Aldrich). The EnVision Plus System-HRP (3,3′-diaminobenzidine, Dako) was used to detect antibody binding. Negative controls were prepared by omitting the primary antibody.

**Figure 3:**
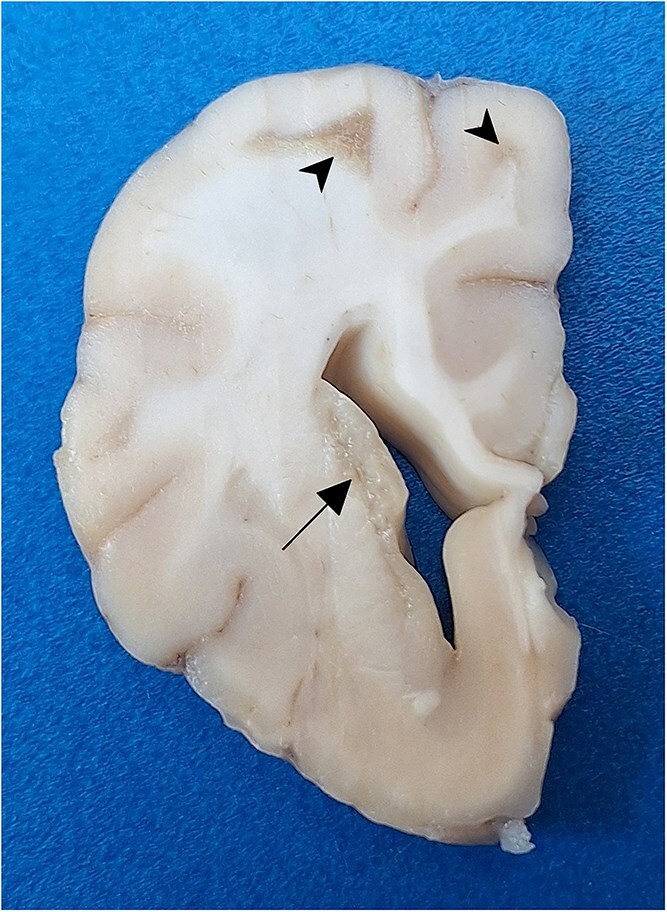
Section of the fixed brain at the level of caudate nuclei. Extensive necrosis and cavitation of the caudate nucleus (arrow) and multifocal necrosis of deep cortical areas (arrowheads).

**Figure 4:**
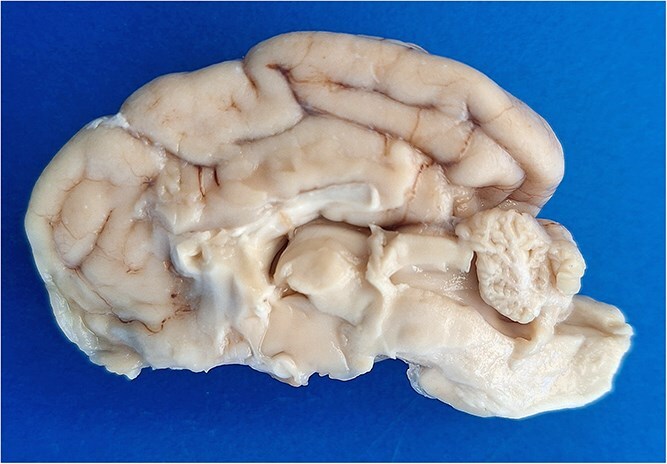
Longitudinal section of the brain. Marked reduction in size of the cerebellum.

Histologically, the malacic areas showed extensive necrosis with post-necrotic cavitation and marked peripheral gliosis (Figure 5). A small necrotic focus was evident in the dorsal thalamus, and multiple necrotic foci, associated with microglial activation and astrocytic reactivity, were detected at the boundary between the subcortical white matter and neocortex (Figure 6). Extensive necrosis and cavitation were also evident in the parahippocampal gyrus and were interpreted as post-ictal lesions. Neurons of the substantia nigra and olivary nuclei were morphologically normal and only slight microvacuolization of the neuropil and scattered swollen axons were observed. In the cerebellum, there was severe atrophy of the cerebellar cortex (Figure 7) with extensive degeneration and a loss of Purkinje cells and, to a lesser extent, of granular and molecular cells (Figure 8), accompanied by moderate reactivity of Bergmann glia. These lesions were suggestive of a multisystemic atrophy consistent with multiple system degeneration (MSD).

**Figure 5:**
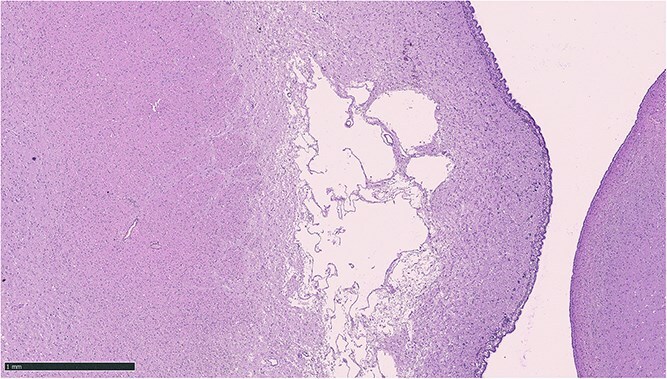
Cavitation of the caudate nucleus. H&E, bar = 1 mm. Abbreviation: H&E = hematoxylin and eosin.

**Figure 6:**
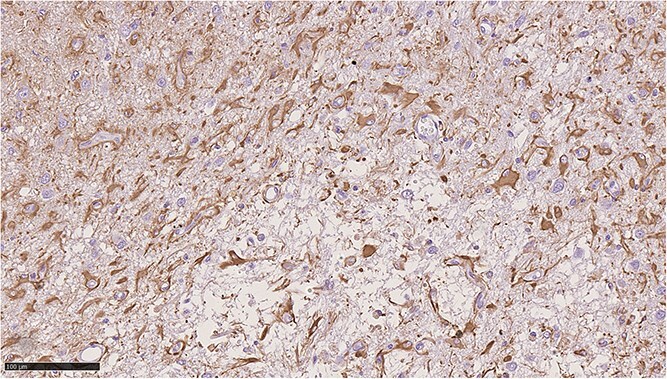
Cerebral cortex. Hypertrophic astrocytes surrounding a spongiotic area. GFAP, bar = 100 μm. Abbreviation: GFAP = glial fibrillary acidic protein.

**Figure 7:**
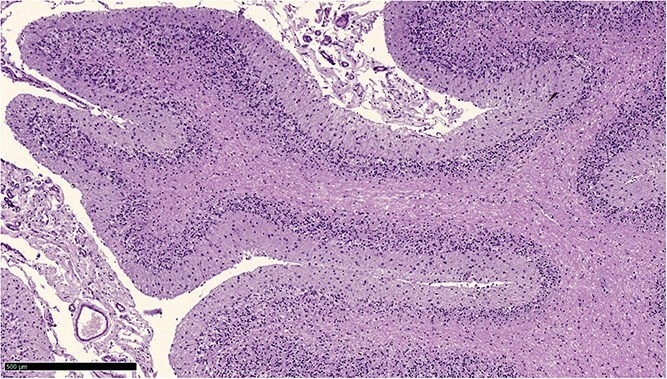
Cerebellar cortex. Atrophy of the folia with widening of the subarachnoid space. H&E, bar = 500 μm. Abbreviation: H&E = hematoxylin and eosin.

**Figure 8:**
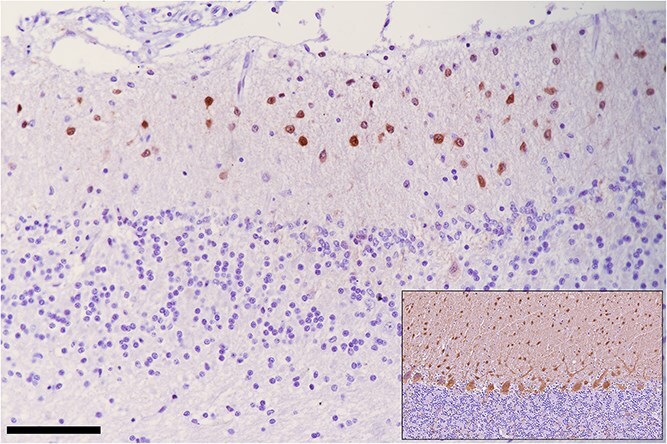
Cerebellar cortex. Extensive loss of Purkinje cells accompanied by moderate depletion of molecular and granule cells. Parvalbumin, bar = 100 μm. Inset: Expression of parvalbumin in a canine normal cerebellar cortex.

Archived EDTA blood samples from the dog were submitted to an external laboratory (Laboklin UK, Manchester) for genetic testing. The dog did not carry any mutant alleles at the previously reported variants in the *SERAC1* gene causing MSD in Kerry Blue Terriers or Chinese Crested dogs (https://omia.org/OMIA001468/9615/). Subsequently, whole-genome sequencing at 26× coverage and filtering for private variants against 1664 control genomes from genetically diverse dogs revealed 31 heterozygous and 13 homozygous variants with a predicted protein-changing effect (Tables S1 and S2). Visual inspection identified a clear top candidate variant in the *SERAC1* gene on chromosome 1. This variant, a single base insertion, can be designated as NC_049222.1:g.48,138,835dupT or XM_038526434.1:c.211dupA. It causes a frameshift truncating almost 90% of the open reading frame, XP_038382362.1:p.(M71Nfs*14). Using the ACMG/AMP criteria from human medicine the variant was classified as likely pathogenic (Table S3).^1^ Relatives of the affected dog were not available for further genetic or clinical investigations.

Clinical, imaging, and neuropathological features, and genetic testing were suggestive of MSD, a progressive inherited neurodegenerative disorder. Multiple system degeneration was first described in Kerry Blue Terriers as “hereditary striatonigral and cerebello-olivary degeneration.”^2–4^ It was later reported in Chinese Crested dogs and reclassified as “canine multiple system degeneration.” Recently, it has also been described in the Ibizan Hound.^3^

Multiple system degeneration in dogs has been linked to loss-of-function variants in the *SERAC1* gene encoding serine active site-containing protein 1. *SERAC1* is located on the outer mitochondrial membrane and plays a crucial role in serine transport, phospholipid composition, and intracellular cholesterol trafficking.^5^^,^^6^ Loss of *SERAC1* function leads to impaired mitochondrial function, and altered energy metabolism is considered central to disease pathogenesis. The disease is transmitted in an autosomal recessive manner.^5^ However, not all canine cases show identifiable *SERAC1* variants, as reported in the Ibizan Hound,^3^ suggesting possible locus heterogeneity. Similarly, in our case, the commercially available targeted genetic tests (validated only for Kerry Blue Terriers and Chinese Crested dogs) failed to confirm a pathogenic variant, whereas whole-genome sequencing identified a new *SERAC1* variant that has not been reported in the literature.

In humans, *SERAC1* variants are associated with 3 phenotypic manifestations: MEGD(H)EL syndrome (3-methylglutaconic aciduria with deafness-dystonia, [hepatopathy], encephalopathy, and Leigh-like syndrome), juvenile-onset complicated hereditary spastic paraplegia, and adult-onset generalized dystonia.^7^ In previous studies, MSD in dogs was closely related to MEGD(H)EL syndrome.^5^

In MSD in dogs, clinical signs usually develop between 3 and 6 months of age^3^^,^^8^ although a later onset has been described, including in a 4-year-old dog.^3^ In human MEGD(H)EL syndrome, 2 forms have been described: an “early infancy” form, presenting at birth with hypoglycemia and failure to thrive, and a “childhood” form, with onset between 2 and 7 years.^9^ In our case, subtle and progressive signs of cerebellar dysfunction were evident during the first year of life.

Cerebellar ataxia, characterized by intention tremors and dysmetria, is described as the initial clinical presentation in dogs with MSD, progressively evolving into frequent falling and an abnormal gait described as “festination.”^8^ Balance loss and stumbling were similarly observed in the dog described herein. In the human literature, a festinating gait refers to short, rapid, hypometric steps, which is a distinctive feature of Parkinson’s disease.^10^^,^^11^ It has been attributed to basal ganglia dysfunction and abnormal motor signaling.^11^ Given the involvement of the basal nuclei in canine MSD, a similar mechanism may be hypothesized.

In the later stages of MSD, postural instability, dystonia, and akinesia occur. Mentation is reported to remain normal in most cases, which may help differentiate MSD from other neurodegenerative disorders.^8^

All human *SERAC1*-related phenotypes are characterized by dystonia, which is also consistent with MSD in dogs.^5^ In MEGD(H)EL syndrome, psychomotor delay, neonatal hypotonia, spasticity, and seizures are common. In the present case, the dog experienced episodic myoclonus with concurrent urination and sialorrhea. Electroencephalography was not performed, so it is unclear whether these episodes were epileptic in origin or movement disorders. However, after the administration of antiseizure medication, no further episodes occurred. Epileptic seizures have not been reported in dogs with MSD. In human medicine, caudate nucleus lesions are associated with movement disorders such as chorea, as observed in Huntington’s disease, where degenerative changes cause a loss of GABAergic neurons and an imbalance between excitatory and inhibitory signaling, resulting in involuntary abrupt movements.^12^^,^^13^ This could potentially explain the myoclonic movements observed in our case.

Bloodwork and ammonia concentrations were normal, consistent with previously described canine cases. Human MEGD(H)EL syndrome is often associated with hyperammonemia, elevated serum lactate concentration, and hypocholesterolemia.^9^ Definitive diagnosis in humans relies on abnormally high urinary 3-methylglutaconic acid or 3-methylglutaric acid concentrations, or the presence of biallelic *SERAC1* variants.^9^ Urine organic acid analysis was not performed in our case.

Magnetic resonance imaging revealed brain and cerebellar atrophy with bilateral caudate nucleus abnormalities, consistent with reported MSD findings. However, unlike previous cases, abnormalities of the substantia nigra and the olivary nuclei were not observed. Cerebral atrophy is not reported in MSD and could represent a novel feature in this case. Bilateral symmetrical lesions are typical of neurodegenerative and metabolic diseases,^14^ and raise a wide differential diagnosis.^15–17^ Notably, bilateral caudate nucleus lesions are also described in Leigh syndrome, a necrotizing encephalomyelopathy of both humans and animals. Although the specific distribution of lesions on the MRI may vary between different forms of Leigh syndrome, it commonly involves the thalamus, rostral and caudal colliculi, and vestibular nuclei.^15^^,^^16^ We do not completely exclude the possibility that MSD in this dog might be a subtype of Leigh syndrome.

Cerebral and cerebellar atrophy is described in neuronal ceroid lipofuscinosis (NCL)^14^; however, the corpus callosum abnormalities reported in NCL were absent in our case, and caudate nucleus involvement is not reported in NCL. Deposits of intracytoplasmic lipofuscin or autofluorescent storage material were not observed.

An atypical presentation of thiamine deficiency with bilateral, contrast-enhancing caudate lesions has been described in a dog.^17^ However, in our case, no contrast enhancement was seen, and thiamine supplementation did not improve clinical signs. Furthermore, cerebellar and cerebral atrophy are not characteristics of thiamine deficiency.^18^

Histopathological findings in our case were mostly consistent with previous descriptions. The combination of cerebellar degeneration and caudate nucleus involvement reflects the lesion distribution of MSD. Reported changes include depletion of the granular and molecular layers, complete Purkinje cell loss,^2–4^^,^^8^^,^^19^ as well as neuronal loss and gliosis in the caudate nuclei.^3^ A lesion pattern has been proposed in which the cerebellar and caudate nuclei are affected first, followed by the substantia nigra and olivary nucleus.^3^^,^^4^ Bilateral and symmetric malacic foci in the dorsal thalamus, as well as multifocal subcortical malacic lesions were also observed microscopically in our dog.

Clinically, early recognition of MSD in dogs remains challenging, particularly in breeds in which the disease has not been reported. Diagnosis relies on a combination of clinical signs, advanced neuroimaging, histopathology, and genetic testing. The prognosis is poor, with euthanasia typically required within 2 years of diagnosis. Currently, no curative treatments are available.

## Supplementary Material

Supplementary_material_aalag141

## Data Availability

Accessions of the whole genome sequence data are given in Table S1.

## Off-label antimicrobial declaration

The authors declare no off-label use of antimicrobials.

## Institutional animal care and use committee or other approval declaration

Dog blood samples from healthy control dogs were collected with the approval of the Cantonal Committee for Animal Experiments (Canton of Bern, Switzerland; permit BE94/2022).

## Abbreviations

MSD: multiple system degeneration
CSF: cerebrospinal fluid
MRI: magnetic resonance imaging
T1W: T1-weighted
T2W: T2-weighted
