# Supplementary material for: Clinical, imaging, and neuropathological characterization of multiple system degeneration associated with a novel SERAC1 variant in a mixed-breed dog
Source: J Vet Intern Med. 2026 Jul 13;40(4):aalag141. doi: 10.1093/jvimsj/aalag141 (PMC13363255; doi:10.1093/jvimsj/aalag141)
Supplement: Supplementary_material_aalag141 [file supplementary_material_aalag141.zip › Video legends.docx]

**Video legends**

Video S1. The gait examination showed cerebellar ataxia characterized by hypermetria on all four limbs.

Video S2. The video shows the affected dog two years after the initial presentation. Neurological examination revealed disorientation and a wide-base stance in the pelvic limbs. The dog exhibits intention head tremors and severe cerebellar ataxia along with frequent loss of balance and falling on either side. Postural reactions and paw replacement test were decreased on all four limbs, worse on the pelvic limbs.
